# Developing a new production host from a blueprint: *Bacillus pumilus* as an industrial enzyme producer

**DOI:** 10.1186/1475-2859-13-46

**Published:** 2014-03-24

**Authors:** Tobias Küppers, Victoria Steffen, Hendrik Hellmuth, Timothy O’Connell, Johannes Bongaerts, Karl-Heinz Maurer, Wolfgang Wiechert

**Affiliations:** 1 Henkel AG & Co. KGaA, Henkelstraße 67, 40589 Düsseldorf, Germany; 2 Fachhochschule Aachen, Heinrich-Mußmann-Str.1, 52428 Jülich, Germany; 3 AB Enzymes GmbH, Feldbergstraße 78, 64293 Darmstadt, Germany; 4 Forschungszentrum Jülich GmbH, Wilhelm-Johnen-Straße, 52428 Jülich, Germany

**Keywords:** Production Strain Blueprinting, *Bacillus pumilus*, Production platform, Expression host, Strain development, Process optimization, Protease, α-amylase

## Abstract

**Background:**

Since volatile and rising cost factors such as energy, raw materials and market competitiveness have a significant impact on the economic efficiency of biotechnological bulk productions, industrial processes need to be steadily improved and optimized. Thereby the current production hosts can undergo various limitations. To overcome those limitations and in addition increase the diversity of available production hosts for future applications, we suggest a Production Strain Blueprinting (PSB) strategy to develop new production systems in a reduced time lapse in contrast to a development from scratch.

To demonstrate this approach, *Bacillus pumilus* has been developed as an alternative expression platform for the production of alkaline enzymes in reference to the established industrial production host *Bacillus licheniformis*.

**Results:**

To develop the selected *B. pumilus* as an alternative production host the suggested PSB strategy was applied proceeding in the following steps (dedicated product titers are scaled to the protease titer of Henkel’s industrial production strain *B. licheniformis* at lab scale): Introduction of a protease production plasmid, adaptation of a protease production process (44%), process optimization (92%) and expression optimization (114%). To further evaluate the production capability of the developed *B. pumilus* platform, the target protease was substituted by an α-amylase. The expression performance was tested under the previously optimized protease process conditions and under subsequently adapted process conditions resulting in a maximum product titer of 65% in reference to *B. licheniformis* protease titer.

**Conclusions:**

In this contribution the applied PSB strategy performed very well for the development of *B. pumilus* as an alternative production strain. Thereby the engineered *B. pumilus* expression platform even exceeded the protease titer of the industrial production host *B. licheniformis* by 14%. This result exhibits a remarkable potential of *B. pumilus* to be the basis for a next generation production host, since the strain has still a large potential for further genetic engineering. The final amylase titer of 65% in reference to *B. licheniformis* protease titer suggests that the developed *B. pumilus* expression platform is also suitable for an efficient production of non-proteolytic enzymes reaching a final titer of several grams per liter without complex process modifications.

## Background

In times of volatile and rising prices of raw materials and energy, high yields are crucial to ensure an economically suitable biotechnological production of bulk products. Additionally, sustainability factors such as substrate availability and low carbon footprint or water consumption are increasing in their importance. Consequently, the production of established biotechnological products needs to be continuously improved in order to ensure sustainable production.

In this context industrial bioprocess development usually proceeds in a series of steps, starting with host selection followed by strain development, process development, downstream processing and scale up. Since time to market is crucial for economic success, the potential of most bioprocesses is not fully realized when production begins. Often such process productivity prematurely runs into a plateau and stays behind the expected performance values. Two clear trends have emerged: 1. process development runs into problems in the late development phase resulting in a delay/termination and 2. a suboptimal choice of production host can lead to a substantial loss of market competitiveness during the production phase also leading to termination.

In each of the described critical situations systematic trouble shooting strategies are required to generate alternatives. A new strategy called Production Strain Blueprinting (PSB) is proposed in the present contribution. The basic idea is based on two different aspects: 1. To model a new production host from the blueprint of an already existing one or at least from a sufficiently high developed lab strain and 2. To select such a new production host in the established production process or a minor variant thereof. Due to the existing experience with the model and production strains and the known process limitations, it can be expected that such an approach will have a comparable if not higher chance of success than classical or modern strain development. Finally, as such a process should lead to a wild-type strain with comparable production characteristics to the established strain, the chances of further improvements are high. PSB provides also the opportunity to develop several strains in a significantly reduced time lapse in order to generate a differentiated production strain library thus allowing for more flexibility in strain choice and production capabilities, all based on the same production process.

The PSB strategy proceeds in the following steps:

I. Screening & Phylogenetic Classification

• Based on the known limitations of the current strain alternative, wild type strains are selected by targeted screening of own resources or public strain collections.

• A sufficiently close genetic relationship to the original production strain must be assured which can be verified by genome sequencing and annotation.

II. Strain Selection & Genetic Toolbox

• Based on a performance test suite, for example by expression capability of native enzymes, one single candidate is chosen for further development.

• An efficient genetic tool box has to be developed or adapted for the chosen strain.

III. Iterative Strain & Process Development

• Relevant genetic variations of the existing production strain are transferred into the new strain in a well planned step by step procedure.

• In each step the process performance is immediately tested in a bioreactor under process operation conditions similar to the established process.

• Process operation conditions are continuously adapted to the new strain.

• At the same time systems-biology-tools including transcriptomics, proteomics, metabolomics and fluxomics approaches may be applied in order to monitor the differences between model and new strain during the iterative development [1].

In order to demonstrate the PSB approach, *Bacillus pumilus* has been developed as a new platform organism for the expression of detergent enzymes by using the established industrial production host *Bacillus licheniformis* as a blueprint. A variant of the subtilisin BL [2] as described in WO9523221 and alkaline α-amylase derived from *Bacillus spec. A7-7* (DSM 12368), both of industrial relevance, were used as reference enzymes. The subtilisin BL variant is named in this contribution as subtilisin BL18. The generated strain variants of *B. pumilus* were characterized in an adapted standard fermentation process under fed batch conditions.

### Production of technical enzymes

Production of technical enzymes is an important part of industrial biotechnology. Alongside their use in paper, leather, textile, bioethanol, food and feed industry, they are used as an essential ingredient of modern detergents reaching a total market volume of about 1.4 billion US-Dollar in 2011 and thereby contributing approximately one third of the total world enzyme market. Alkaline and high-alkaline proteases are the most prominent detergent enzymes and contribute – alone or in combination with α-amylases – to the basic performance of modern detergents. Such proteases already reached in 2002 an annual tonnage of about 900 metric tons equivalent of pure enzyme for the European market, tendency increasing [3].

As industrial enzymes used in white biotechnology mostly have to be considered as commodities, the production costs have to be, comparatively, very low. Several grams per liter are needed for an economical production. Therefore, an enhancement of the production yield is an important factor of every production process, even for well established products.

Species of the gram-positive, spore forming genus *Bacillus* belong to the most important expression hosts for the industrial enzyme production [4-7], with reported extracellular enzyme yields up to 20-25 g/l [8].

Over the last decades, organisms such as *Bacillus alkalophilus, Bacillus halodurans, Bacillus lentus, Bacillus licheniformis* or *Bacillus subtilis* have been used to manufacture alkaline and high alkaline enzymes in large scale industrial production as compounds in for example washing and cleaning agents. Nevertheless, it is important to identify and develop new production hosts to improve the availability of industrial enzymes, both novel and existent, in competitive yields and to open the process for new potential.

The screening of different *Bacillus* species is a promising route for the development of better production systems. For example, *Bacillus megaterium* has already been investigated as a potential production host [9,10]. Another interesting candidate, *Bacillus pumilus*, is used in this contribution.

A particular challenge for the evaluation of new enzyme production hosts is the fact that industrial fermentation is a complex process, which has to be aligned to the production system as well as to the designated product. Therefore, even if a production host with a potential to secrete a desired enzyme in high yields has been found, it is still a complicated task to establish a fermentation process with industrial relevance.

Despite of metabolic models being state of the art for improving small molecule productions [11,12], reliable mathematical models have not yet been established for the production of complex proteins. Thus in this work, we have not applied system biological approaches during the development of an efficient alternative production host.

## Results and discussion

Our aim was to demonstrate that the suggested PSB strategy can be applied successfully to develop a new production host with higher yields based on an already established production process. This requires only minor adaptations compared to the long lasting development needed if started from scratch. Therefore, we developed *B. pumilus* for the production of industrial enzymes, as shown in case of the subtilisin BL variant (BL18) and A7-7 s α-amylase, starting with a fermentation process developed for *B. licheniformis*.

In this work, we focused especially on developing the production platform in the described iterative procedure. The development of the genetic toolbox as well as the characterization of *B. pumilus* Jo2 under various environmental conditions by proteomic (and transcriptomic) approaches are subject of the BMBF (*Bundesministerium für Bildung und Forschung*) funded project “Microbes for production: A genomics-based approach to engineer novel industrial production strains” and will be published elsewhere.

To demonstrate our approach in the following, we want to focus especially on item III of the PSB strategy. Therefore only some basic facts which are relevant for the further understanding are summarized in items I and II.

I. Screening & Phylogenetic classification

In a preliminary screening, the synthesis and secretion capability of homologous extracellular proteases was taken as decisive parameter. Therefore various adherent growing *Bacillus species* from the DSMZ and Henkel strain collections were pre-selected based on their proteolytic activity as observed on a skim milk based plate assay.

To be able to assess the phylogenetic relationship between the established production host *B. licheniformis* and the best hits of the screened *Bacilli species*, best hits were evaluated by whole genome sequencing as well as phylogenetic classification based on their biochemical properties, fatty acid profile and 16S-rDNA. Some examples of such best hits identified are *B. pumilus* or alternative *B. licheniformis* strains.

II. Strain selection & genetic Toolbox

In addition to the phylogenetic classification and genetic relationship to the current production strain, promising strains were furthermore assessed for their known pathogenic factors, genetic accessibility and genetic make-up. The genetic accessibility, especially the potential barrier of restriction modification systems by DNA methylation, was one hurdle to ensure a fast strain development. Additionally the genetic make-up of the different strains had to be considered as well. In regard to these parameters the *B. pumilus* strain Jo2 (Jo2) was selected because of its high proteolytic activity, its genetic-accessibility, its native ability to produce extracellular enzymes and its relatively small genome (approx. 0.5 Mbp smaller than the genome of known *B. subtilis* or *B. licheniformis*). Despite of Jo2 having natively six extracellular proteases (A*pr*E1, A*pr*E2, M*pr*, E*pr*; V*pr*, B*pr*), the strain has in contrast to *B. subtilis* as well as *B. licheniformis* no chromosomally encoded amylase. In addition to the absence of a natural Amylase the presence of two subtilisins (A*pr*E1, A*pr*E2) is, in contrast to other *Bacillus spec*., quite unusual.

Additionally to the adapted cloning methods used in this contribution, the development of a genetic toolbox for genetically engineering Jo2 was realized and described by Wemhoff and coworkers [13]. The spectrum of adapted and developed methods assures a straight forward strain development and allows an efficient genetic engineering of Jo2.

III. Iterative strain and process development

To evaluate and improve Jo2’s production capability of extracellular enzymes, the strain as well as the fermentation process was optimized as described above in an iterative procedure to obtain improved fermentation yields. Therefore genetic modifications were introduced step by step into *B. pumilus* in reference to the production strain *B. licheniformis* and other genetic modifications described elsewhere. Simultaneously, a *B. pumilus* fermentation process was developed and optimized at lab scale based on downscaled production process of the current production host *B. licheniformis*.

### Initial strain modification

*B. pumilus* spores are highly resistant against oxidative as well as thermal stress and thereby cause significant difficulties in sterilization procedures [14,15]. Thus, as a first step the endospore formation was knocked out in *B. pumilus* strain derivative Jo2.1 [13]. As with *B. licheniformis*, where the knocking out of the endospore formation has been described previously by Nahrstedt *et. al.*[16], the gene *yqf*D (*spoIV* Jo2 homolog) was deleted in the chromosome resulting in a strain with inhibited spore germination, Jo2.1 [13]. In this contribution Jo2.1 is the host which was used in bioreactor cultivations to avoid spore contamination.

For a detailed characterization in regard to *B. pumilus’* production capability of extracellular enzymes the well characterized detergent Protease subtilisin BL18 was chosen as a reference molecule. To overexpress the target molecule its gene was introduced into the strain background Jo2.1 as part of a stable and self-replicating production plasmid pHP49 (Henkel AG & Co. KGaA) derived from pBC16 [17]. Besides of the pBC16 backbone it contained an expression cassette consisting of a protease promoter, defined as reference (P_ref_) in this contribution, a sec-pathway dependent signal peptide and the mature subtilisin BL18 protease gene with its upstream located propeptide, involved in the extracellular folding of the secreted protease [18]. As with any other plasmid in this contribution the *in vitro* methylated plasmid DNA was introduced into Jo2.1 by PEG mediated protoplast transformation to thereby overcome the barrier of *B. pumilus* Jo2.1’s restriction modification system.

### Process adaptation and optimization

To ensure the comparability of the bioreactor based process for the comparison of strain derivatives, the initial process was carried out in a “close-to-production” fed batch process according to an established production protocol for the host *B. licheniformis*. Subsequent process optimization was then carried out to adapt the process for Jo2.1. The achieved protease concentration in the culture supernatant of *B. pumilus* cultivations was normalized to the protease titer of Henkel’s industrial production strain *B. licheniformis* carrying the plasmid pHP49 (Henkel AG & Co. KGaA), serving as reference of the established production host cultivated in the same scale.

**Figure 1:**
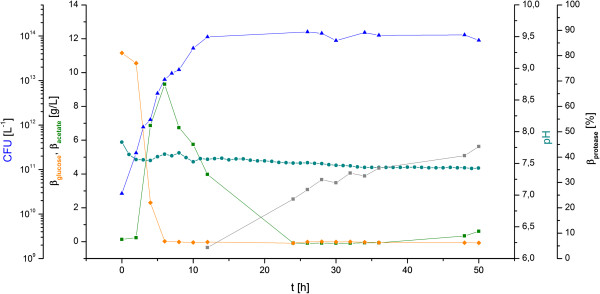
**Fed batch cultivation of *B. pumilus *Jo2.1/pHP49 at lab scale.** Adapted cultivation process based on a down-scaled production process of *B. licheniformis*. Colony forming Units (CFU) (blue triangle) [L^-1^], glucose concentration (orange diamond) [g/l], acetate concentration (green square) [g/l] and protease titer (gray square) were measured at line. The pH-value (light blue circle) was monitored online. Yielded enzyme concentrations scaled in correlation to the subtilisin BL18 protease titer of the current production host *B. licheniformis* in a lab scale cultivation serving as industrially relevant reference in this contribution.

As shown in Figure 1, the curves of the different parameters are quite comparable to those of *B. licheniformis* cultivations for protease production as described previously [19-21]. Biomass is formed during the batch phase and the initial fed batch phase within the first 10 to 12 h with an increasing cell count up to approximately 2.3*10^13^ CFU/L. Glucose is consumed as the primary carbon source during the initial growth phase while the formation of overflow metabolites is observed. In addition to acetate, the most significant overflow metabolite, the accumulation of other side products, such as acetoin and 2,3-butandiol were observed, which were measured in the supernatant (data not shown) and previously described for *B. pumilus* cultivations [22].

Based on the acetate concentration reaching its peak value at the same time while the glucose is completely depleted and in correlation with the rapidly inclining dissolved oxygen (DO) concentration (data not shown), the culture displayed a diauxic behavior. This leads to a metabolic change towards the incipient consumption of acetate as alternative carbon source, as indicated by the subsequently declining acetate concentrations in the supernatant.

When the diauxie occurred, the glucose feed was initiated with an average feeding rate of 2.13 g/(L*h). pH was set and maintained at a constant value of 7.50 ± 0.15. The accumulation of the extracellular target protease was measurable after approximately 12 h, showing a continuous increase in protease concentration over time and reaching a maximum titer of about 44.2% in comparison to the established production strain *B. licheniformis*.

**Figure 2:**
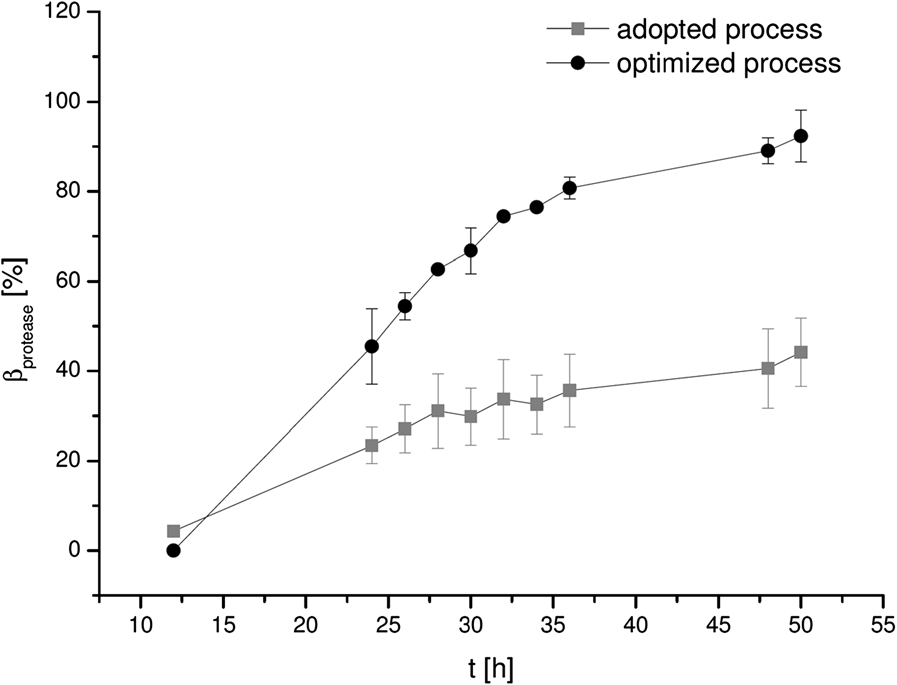
**Protease titer of *B. pumilus *Jo2.1/pHP49 cultivations under adopted (gray square) and optimized (black circle) process conditions at lab scale.** Yielded enzyme concentrations scaled in correlation to the subtilisin BL18 protease titer of the current production host *B. licheniformis*/pHP49 in a lab scale cultivation serving as industrial relevant reference in this contribution. Error bars represents the standard deviation of three biological replicates.

To optimize the fermentation process and to adopt it to the current strain Jo2.1/pHP49 the influences of various process parameters were examined in order to achieve a higher product yield. The modification of the glucose feeding rate as well as the pH-profile under current process conditions was proven to contribute significantly to an improved product yield. Therefore process modifications were introduced to avoid acetate accumulation at the end of the process by an adjusted glucose feeding as well as to improve the stability of the active target enzyme in the culture supernatant by shifting the pH towards the stability-optimum of the target molecule during the production phase. The peak value under both process setups was reached at the end of the process with its maximum of 44.2 ± 7.6% under adapted and 92.3 ± 5.8% under optimized process conditions, respectively (Figure 2). Herby it is shown, that the improvement of these two parameters pH-profile and C-feeding led to a largely increased product formation rate during the product accumulation, resulting overall in 2.25-fold protease titer.

### Plasmid backbone validation

To validate and thereby optimize *B. pumilus* as production host the influence of the plasmid on the expression capacity was investigated. Therefore, the expression capacity using the current plasmid backbone of pHP49 was compared with the expression performance using three other production plasmids (pHP17, pHP59, pPB49), which were selected out of Henkel´s plasmid toolbox. The three plasmids pHP17, pHP59, pPB49 and the already used plasmid pHP49 differed in the length as well as the sequence of their specific backbone, but not in their protease expression cassette. The plasmid backbones of pHP49 and pHP59 are based on plasmid pBC16 [17], the backbones of pPB49 and pHP17 on plasmid pUB110 [23,24], respectively. The tetracycline resistance gene of the pBC16 derived plasmids was previously replaced by the pUB110 kanamycin resistance gene. Hence, all plasmids confer kanamycin resistance to the host strains. The backbones vary in size (pHP49 vs. pHP59 and pPB49 vs. pHP17, respectively) since in pHP59 and pHP17 non-relevant regions (e.g. truncated *mob* gene) were removed. Plasmids were *in vitro* methylated and introduced into Jo2.1 cells by protoplast transformation.

Jo2.1 strains containing the four different backbones were comparatively cultivated under optimized process conditions. The different plasmid backbones led to variations in the specific product formation rate during product accumulation as well as to significantly differing maximum protease concentrations. The pBC16 based plasmids pHP49 and pHP59 reached a value of 92.3 ± 5.8% and 80.3 ± 2.4%, respectively. In comparison, both pUB110 derived plasmids pPB49 and pHP17 reached a final score of 78.8 ± 0.6% and 60.4 ± 0.8%, respectively. Interestingly, in both cases the plasmids with the reduced backbone size (pHP59, pHP17) showed the lower protease production. The cultivations of the pUB110 based plasmids were only performed in duplicate, in contrast to other cultivations in triplicate at least. Thus, the very high reproducibility as indicated by the low deviation from their arithmetic average in conjunction with their highly comparable process data qualified these processes as a sufficient reference. Overall, the highest protease titer was still reached in Jo2.1/pHP49 cultivations with the value of approximately 92.3 ± 5.8%. Therefore, the plasmid pHP49 was selected for further promoter studies.

### Promoter optimization

As extracellular proteases belong to the most prominent proteins in the secretome of *B. licheniformis*[25] and *B. pumilus* (data not shown) during stationary growth phase, the homologous promoters of the *B. pumilus* Jo2 chromosomal proteases *apr*E1, *apr*E2 and *mpr* were selected for the subsequent promoter optimization approach. Based on the previous results, the pHP49 backbone was selected and its current promoter P_ref_ was substituted by four variants of the homologous promoter P_*apr*E1_ (pVS13, pVS14, pVS23, pVS24) as well as the promoters P_*apr*E2_ (pVS20) and P_*mpr*_ (pVS19) of *B. pumilus* Jo2 using enzyme free cloning. The different variants of the homologous aprE1 promoters were generated by successively shortening the promoter sequence in order to optimize the expression performance. The lengths of the resulting promoter sequences are as follows: P_*apr*E1 I_ 555 bp, P_*apr*E1 II_ 380 bp, P_*apr*E1 III_ 357 bp, and P_*apr*E1 IV_ 351 bp. All promoter fragments were PCR amplified from chromosomal template DNA and introduced into the pHP49 plasmid backbone by enzyme free cloning. Constructed plasmids were introduced into Jo2.1 and the newly generated strains were cultivated under the previously obtained optimized process conditions at lab scale.

**Figure 3:**
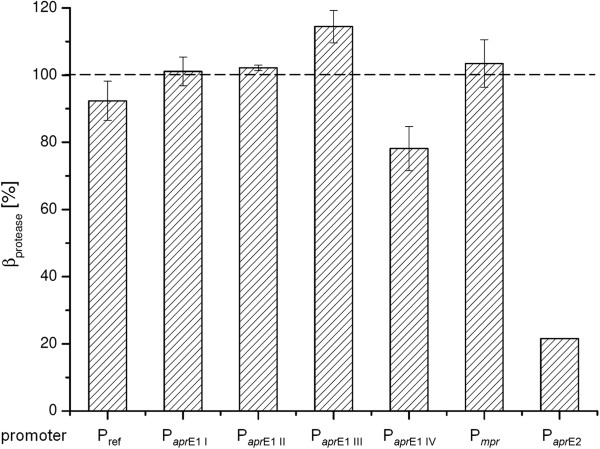
**Promoter optimization in the pHP49 backbone for improving the protease titer in *B. pumilus *Jo2.1 cultivations.** Used promoters: P_ref_, P_*apr*E1 I_, P_*apr*E1 II_, P_*apr*E1 III_, P_*apr*E1 IV_, P_*mpr*_ and P_*apr*E2_. Cultivations were carried out in the optimized fed batch fermentation process. Yielded enzyme concentrations scaled in correlation to the subtilisin BL18 protease titer of the current production host *B. licheniformis*/pHP49 in a lab scale cultivation serving as industrial relevant reference in this contribution. Error bars represents the standard deviation of at least three biological replicates. P_*apr*E2_ represents the mean value of only two independent cultivations (with this promoter the two fermenter yields were essentially identical).

As shown in Figure 3, the promoters P_*apr*E1 I_, P_*apr*E1 II_ and P_*mpr*_ reached an average protease titer quite comparable to the industrial production host of approximately 100%. In contrast to these results the shortened promoter P_*arp*E1 IV_ resulted in a reduced protease titer of about 78.1 ± 6.6%, which is especially under consideration of the shortened promoter variant P_*arp*E1 III_ a remarkable poor result. Due to a lowered protease activity in P_*apr*E2_ cultivations the average protease titer of two almost identical replicates reached a maximum of only 21.6%. Depending on the congruence of these two independent replicates in conjunction with its poor production performance no further characterization of P_*apr*E2_ was carried out. The highest expression capacity was achieved by derivate III of the homologues P_*apr*E1_ promoter (P_*apr*E1 III_) reaching an average value of 114.5 ± 4.8%, and thereby accomplished an increase of 14.5% of the protease production in comparison to the established production host.

### Evaluation of the Jo2.1 expression platform as a production host for non proteolytic hydrolases

As *B. pumilus* Jo2.1 showed a remarkable potential in synthesizing and secreting the extracellular protease subtilisin BL18 with up to 114.5% in comparison to the established production host at lab scale, it was conjectured that the expression system under control of the protease promoter could be used to overexpress alternative non-protease target enzymes efficiently. Thus, the alkaline α-amylase of *Bacillus spec*. A7-7 was exemplarily selected as an industrial relevant non-protease enzyme to substitute the plasmid encoded target enzymes subtilisin BL18 for testing its production capability. The amylase expression plasmid pHP5-31, differing from pHP49 in its encoded target enzyme, A7-7 α-amylase instead of serine protease subtilisin BL18, was introduced into Jo2.1 as described above.

**Figure 4:**
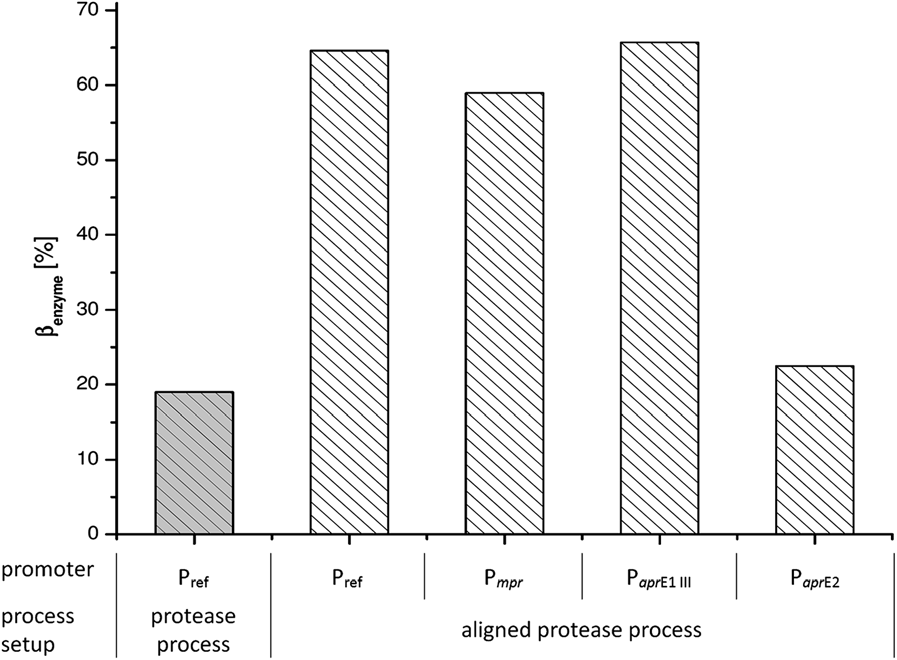
**Maximum amylase titer of *B. pumilus *Jo2.1 cultivations deploying various promoters in the pHP49 backbone.** Jo2.1/pHP5-31 (P_ref_) was initially cultivated under optimized protease process conditions (bar with grey background). Strains containing the plasmid encoded promoter P_ref_, P_*mpr*_,P_*apr*E1 III_ and P_*apr*E2_ were cultivated under aligned process conditions in respect to modified pH setpoint (bars with white background). Based on a harshly decreased process robustness expressing the amylase in the protease process, bars represent the maximum achieved amylase titer of four independent cultivations per strain. Yielded amylase concentrations are scaled to *B. licheniformis* protease BL18 titer at the same scale serving as industrial relevant reference.

The following cultivation of Jo2.1/pHP5-31 under optimized protease process conditions yielded approximately 19.0% active enzyme (Figure 4) in comparison to *B. licheniformis* subtilisin BL18 expressions by using the pHP49 production plasmid in a lab scale cultivation serving as industrial relevant reference in this contribution.

Under given process conditions the pH was identified as the critical parameter in an optimization approach. For this reason the pH was re-adjusted to a constant setpoint of 7.70 ± 0.15 resulting in a significantly increased amylase accumulation in the culture supernatant of approximately 65%. Due to the fact that the process robustness decreased under current process conditions in comparison to protease expressions, the best fermentation of each strain was chosen instead of an average value to assess the different strains in the overexpression of the A7-7 α-amylase. In order to achieve a further improved amylase titer, the homologues protease promoters P_*apr*E1 III_ (pTK6), P_*mpr*_ (pTK5) and P_*apr*E2_ (pTK7) were introduced into pHP5-31 replacing the P_ref_ promoter. Under modified pH conditions the tested promoters P_*apr*E1 III_, P_*mpr*_ and P_*apr*E2_ showed the same trend as in the protease expression cultivations reaching overall a maximum amylase concentration of approximately 65.7% (P_*apr*E1 III_).

## Conclusions

**Figure 5:**
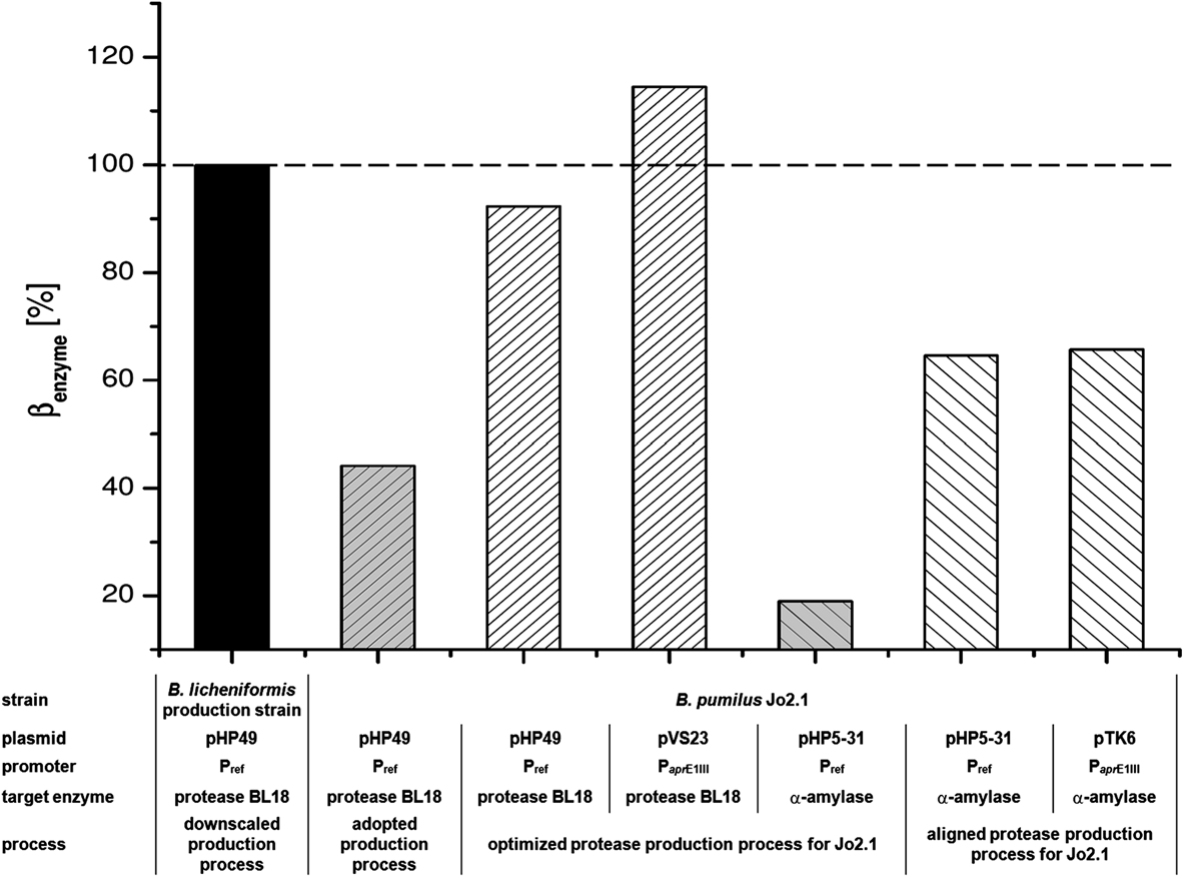
**Maximum enzyme titer of *B. pumilus *Jo2.1 cultivations in reference to the current production host *B. licheniformis *at lab scale: Overexpression of subtilisin BL18 (*B. licheniformis*) vs. subtilisin BL18 (*B. pumilus *Jo2.1) and A7-7 α-amylase (*B. pumilus *Jo2.1), respectively.** Process conditions and specific plasmid configurations are given below the figure. Based on a harshly decreased process robustness expressing the amylase, bars represent the maximum achieved amylase titer of four independent cultivations per strain. Yielded amylase concentrations are scaled to *B. licheniformis* protease titer at the same scale serving as industrial relevant reference for enzyme production.

In this work it is shown for the example system *B. pumilus* that the proposed fast-track-blueprinting-strategy can indeed be applied very efficiently to develop an alternative production host. The presented results were achieved within approximately one year clearly indicating the reduced time lapse to generate new promising production strains. During the presented step by step optimization procedure involving strain as well as process development the chosen *B. pumilus* strain Jo2.1 achieved an increasing protease titer of 44% by process adoption, 92% by process optimization and 114% by the final promoter optimization in comparison to Henkel’s established production strain *B. licheniformis* (Figure 5).

Using a modified protease fermentation process the *B. pumilus* strain Jo2.1/pTK6 reached a maximum amylase titer of approximately 65% in reference to *B. licheniformis* protease titer at the same scale. This titer is significantly lower than the maximum protease titer but indicates in regard to its final product concentration of >4 g/l and under consideration of the insufficiently optimized fermentation process still a remarkable potential of the current *B. pumilus* Jo2.1 for the production of extracellular enzymes.

Moreover, this is to our knowledge the first time that a protease promoter is used to overexpress a non-protease enzyme while yielding such high product titers. Thus, this expression platform exhibits versatility in the spectrum of enzymes it can be used to produce. This versatility resulted from the fact that with the same promoter and only minor process modifications allow for very different enzymes to be produced.

This is in contrast to many systems where changing the target enzyme frequently calls for a switch of the applied promoter resulting in complex process modifications.

In conclusion, these findings demonstrate that the fast-track blueprinting strategy is an interesting alternative in contrast to starting the development from scratch all the more if sufficient information regarding related production hosts is available. Furthermore, the presented *Bacillus* species *B. pumilus* is a very promising candidate as a future production host.

## Methods

### Bacterial strains and plasmids

Bacterial strains and plasmids used in this study are listed in Table 1, oligonucleotides for cloning in Table 2. For details on plasmid constructions refer to Additional file 1 (Appendix S1 and Table S1).

**Table 1:** Bacterial strains and plasmids used in this contribution

**Strain/Plasmid**	**Description**	**Reference**
**Strains**		
*B. subtilis* DB104	*his nprR*2 *nprE*18 Δ*aprA*3	Kawamura & Doi [26]
*B. pumilus* Jo2	Wild type	Henkel AG & Co. KGaA
*B. pumilus* Jo2.1	Jo2 Δ*yqf*D	Wemhoff *et al*. [13]
**Plasmids**		
pHP49	pBC16, P_ref_, subtilisin BL18, Km^R^	Henkel AG & Co. KGaA
pHP59	pBC16*, P_ref_, subtilisin BL18, Km^R^	Henkel AG & Co. KGaA
pPB49	pUB110, P_ref_, subtilisin BL18, Km^R^	Henkel AG & Co. KGaA
pHP17	pUB110*, P_ref_, subtilisin BL18, Km^R^	Henkel AG & Co. KGaA
pVS13	pHP49, but P_*apr*E1 I_	This work
pVS24	pHP49, but P_*apr*E1 II_	This work
pVS23	pHP49, but P_*apr*E1 III_	This work
pVS14	pHP49, but P_*apr*E1 IV_	This work
pVS19	pHP49, but P_*mpr*_	This work
pVS20	pHP49, but P_*apr*E2_	This work
pHP5-31	pBC16, P_ref_, α-amylase, Km^R^	Henkel AG & Co. KGaA
pTK5	pHP5-31, but P_*mpr*_	This work
pTK6	pHP5-31, but P_*apr*E1 III_	This work
pTK7	pHP5-31, but P_*apr*E2_	This work

See Additional file 1 for cloning details (*non-relevant backbone regions deleted).

**Table 2:** Oligonucleotides used in this study

**Oligonucleotide**	**Sequence**
P-pVS13_for_long	CAGCGTGTAGACAAACCTTCGCATTC
P-pVS13_for_short	CCTTCGCATTCGTTGTCAGGTCTGC
P-pVS13/14_rev_long	TCCACATCCCTTTTTTCTTATTTCAGAATAATCATC
P-pVS13/14_rev_short	TCTTATTTCAGAATAATCATCCGTAGTCTATAAGAATG
P- pVS13/14_RG_for_long	AAAAAGGGATGTGGAATGATGAGGAAAAAGAGTTTTTG
P- pVS13/14_RG_for_short	ATGATGAGGAAAAAGAGTTTTTGGCTTGGGATGC
P- pVS13/14_RG_rev_short	TTGCTCAAAAAAATCTCGGTCAGATGTTACTAGCAACTC
P- pVS13_RG_rev_long	TTTGTCTACACGCTGTTGCTCAAAAAAATCTCGGTCAG
P- pVS14_for_long	ATGACAAAAACAATGATAAAATAATATTTTTTTATATCG
P- pVS14_for_short	ATAAAATAATATTTTTTTATATCGAAATTCGAAATAGCTGC
P- pVS14_RG_rev_long	CATTGTTTTTGTCATTTGCTCAAAAAAATCTCGGTCAG
P-pVS19_for_long	AGCAACTGGATCTAACAAGAGGAAAGGCCGCC
P- pVS19_for_short	TAACAAGAGGAAAGGCCGCCAATTAG
P- pVS19_rev_long	TTTTCCTCATCATCATATTCCTCCTTTATGTCCTATATCAAAAATC
P- pVS19_rev_short	CATATTCCTCCTTTATGTCCTATATCAAAAATCATACG
P-pVS18_RG_for_long	ATGATGAGGAAAAAGAGTTTTTGGCTTG
P-pVS18_RG_for_short	AGAGTTTTTGGCTTGGGATGCTGAC
P-pVS18_RG_rev_long	GATCCAGTTGCTCAAAAAAATCTCGGT
P-pVS18_RG_rev_short	CAAAAAAATCTCGGTCAGATGTTACTAGCA
P-pVS20_for_long	AGCAACTGGATCCGAGAACATCTTGAAAGGCA
P-pVS20_for_short	CGAGAACATCTTGAAAGGCAGCACAGC
P-pVS20_rev_long	TTTTCCTCATCATAATACCCACTCTCCCTTTCATCTTTTTGTC
P-pVS20_rev_short	AATACCCACTCTCCCTTTCATCTTTTTGTC
P-pVS23_for	GAGCAATTTTAAATGACAAAAACAATGATAAAATAATATTTTTT
P-pVS23_for_short	ACAAAAACAATGATAAAATAATATTTTTTTATATCGAAATTCGAAATAG
P-pVS23_rev	CATTTAAAATTGCTCAAAAAAATCTCGGTCAGATG
P-pVS23_rev_short	AAAAAAATCTCGGTCAGATGTTACTAGCAACTCA
P-pVS24_for	CTGTTATATAAACAGGTTCTTTTAAATGACAAAAACAATG
P-pVS24_for_short	AGGTTCTTTTAAATGACAAAAACAATGATAAAATAATATTTTTTTATATCG
P-pVS24_rev	GTTTATATAACAGGTTCTTGCTCAAAAAAATCTCGGTCAGATG
P-pVS24_rev_short	GTTCTTGCTCAAAAAAATCTCGGTCAGATG
pHP5-31_backbone_for_long	ATGACGATGAGAAAACGTAAAAATGG
pHP5-31_backbone_for_short	GTAAAAATGGATTAATCAGTATTCTATTGGC
pHP5-31_backbone_rev_long	CATTTACAAGAACAGCATCTTTCCTCG
pHP5-31_backbone_rev_short	CATCTTTCCTCGTTTTTCTTGTACCTG
pTK5/6/7_Insert_for_long	CTGTTCTTGTAAATGAGTTGCTAGTAACATCTG
pTK5/6/7_Insert_for_short	AGTTGCTAGTAACATCTGACCGAGATTTTTTTGAGC
pTK5_Insert_rev_long	GTTTTCTCATCGTCATCATATTCCTCCTTTATGTC
pTK5_Insert_rev_short	CATATTCCTCCTTTATGTCCTATATCAAAAATC
pTK6_Insert_rev_long	GTTTTCTCATCGTCATTCCACATCCCTTTTTTC
pTK6_Insert_rev_short	TCCACATCCCTTTTTTCTTATTTCAGAATAATC
pTK7_Insert_rev_long	GTTTTCTCATCGTCATAATACCCACTCTCCCTTTCATC
pTK7_Insert_rev_short	AATACCCACTCTCCCTTTCATCTTTTTGTC

The sporulation deficient strain variant *B. pumilus* Jo2.1 (Jo2Δ*yqf*D) described by Wemhoff *et al. *[13] was used for all strain development and characterization properties.

Due to *B. pumilus* Jo2’s type I restriction modification system, *B. subtilis* DB104 [26] was used for cloning experiments and yielded high quantities of plasmid DNA for subsequent *in vitro* methylation and transformation of *B. pumilus* cells.

### Molecular and microbiological techniques

Unless stated otherwise, all cultivations in shaking flasks were carried out at a shaking frequency of 280 rpm (ø_shaking_ = 50 mm) with a filling volume not exceeding one tenth of the total flask volume to avoid oxygen limitation [27].

For microbiological and molecular biological applications, cultivations of *B. pumilus* and *B. subtilis* were carried out at 37°C in Luria–Bertani (LB) broth (10 g/l peptone, 5 g/l yeast extract, 10 g/l NaCl) and LB-agar (agar 10 g/l), respectively.

Plasmid and chromosomal DNA of *B. pumilus* cells were extracted by using the QIAprep Spin Miniprep Kit (Qiagen, Hilden, Germany) and nexttec™ DNA isolation kit (nexttec GmbH, Leverkusen, Germany), respectively and the respective methods.

Amplifications of DNA fragments were performed by PCR according to the manufacturer’s recommendation using DNA polymerase Phusion II and Fire I (New England Biolabs).

Analyses of restriction digested plasmid DNA and PCR products were carried out via agarose gel electrophoresis. To extract DNA fragments after gel electrophoresis, the Qiaquick® Gel extraction Kit (Qiagen, Hilden, Germany) was used with the respective method.

PCR products were purified using Qiagen PCR purification Kit (Qiagen, Hilden, Germany) with the respective method.

Recombinant DNA work was carried out using conventional techniques as described by Sambrook & Fritsch [28].

Plasmids were constructed by the PCR based “enzyme free cloning method” as described elsewhere [29,30], using the primers listed in Table 2.

Nicked circular products constructed by the enzyme free cloning method as well as whole plasmids were introduced into *B. subtilis* DB104 and *B. pumilus* Jo2.1 protoplasts in a PEG mediated transformation method as previously described [31].

### Screening of colonies

Extracellular amylase and protease activities were detected on LB-agar by a clear halo surrounding the bacterial colonies containing active enzyme as described by Nahrstedt and coworkers [16]. Before screening plates of amylase containing colonies were replicated according to the method of Lederberg *et al.*[32] for the detection assay. For Protease or amylase activities the LB-agar nutrient was supplemented with 2% skim milk or 1% soluble starch, respectively. In case of the amylase assay, the agar was overlaid with Lugol’s solution to visualize the halo.

### Preculture

Precultures were prepared in 1 L shaking flasks in complex protein medium, containing insoluble plant protein 25 g/l, yeast extract 7.4 g/l and NaCl 5.6 g/l, at 37°C for 10 h. After the cultivation was stopped, 50% (w/V) glycerol solution was added up to a final concentration of 10% (w/V) glycerol. The glycerol containing precultures were aliquoted and stored at -80°C for further use. Precultures were analyzed by microscopy and colony forming units (CFU) were determined on agar plate to check for contamination.

### Fermentation process

Cultivations were carried out in stirred tank reactors (STR) with a 3.2 L total volume (“KLF”, Bioengineering, Switzerland) in a complex medium in deionized water containing: glucose, 15 g/l; complex plant protein, 50 g/l; (NH_4_)_2_SO_4_, 2.8 g/l; KH_2_PO_4_, 6.8 g/l; MgSO_4_*7H_2_O, 1.4 g/l; CaCl_2_*2H_2_O, 0.5 g/l; MnSO_4_*H_2_O, 0.09 g/l; Kanamycin 0.05 g/l; at 39°C, aeration rate of 1.4 vvm. The baffled STR was equipped with pH, dissolved oxygen (DO) and temperature probes for online monitoring and regulation of pH and temperature and DO using stirrer speed. The concentration of O_2_ and CO_2_ in the off-gas analysis was measured by the gas analyzer Siemens Ultramat 23 (Siemens AG, Munich, Germany). Polypropylene glycol with a Mr of 2000 (PPG 2000) was added on demand as an antifoam agent. Unless otherwise stated, all cultivations were carried out with at least three independent cultivations. Error bars represent the standard deviation between the independent cultivations.

### Analytics

The number of viable cells (colony forming units, CFU) in the complex medium was carried out according to aerobic plate count method as described in the bacterial analytical manual by the Food and Drug Administration (FDA) [33]. A set of serial dilutions of 100 μL sample to 900 μL saline solution to a dilution factor of 10^9^ were plated separately on LB-Agar plates and incubated for 24 h at 37°C. The number of viable cells in each sample was estimated based on the average cell count of the dilution rate showing 20 to 250 colonies on the respective agar plates.

Acetate and glucose analytics were carried out in three technical replicates by using the automated photometric analyzer system Arena 30 (Thermo Fisher Scientific GmbH, Dreieich, Germany).

The commercially available assays Enzytec™ fluid D-Glucose and the Enzytec™ fluid acetic acid (Thermo Fisher Scientific GmbH) were applied to measure the glucose and acetate concentrations in culture supernatants.

Protease activity in the culture supernatant was determined by a protease assay described by van Raay *et al*. [34] based on the peptidolytic cleavage of casein. After a specific reaction time, the proteolyses of casein was stopped by adding tri-chloro acetic acid to precipitate the remaining protein which was subsequently separated by centrifugation. The absorbance of aromatic tryptophan side chains in the supernatant was measured at 290 nm and corrected against a blank measurement: 0.5 abs ≙ 10 U/mL. The yielded enzyme activity of the target protease subtilisin BL18 was quantitatively determined by conversion with its specific activity.

The amylase activity in culture supernatants was measured using a colorimetric assay quite similar to ethyliden-pNP-heptaglycosid based α-amylase assay described by Kruse-Jarres *et al. *[35], which is also available in commercial α-amylase assays as for instance alpha-Amylase EPS Pancreatic (Roche Diagnostics GmbH) or Amylase (PNP) Reagent (Fisher Diagnostics). In contrast to the commercial available assay systems, pNP-heptaglucosid carrying a benzyliden protecting group was used instead of ethyliden. Therefore, the reaction mechanism was slightly modified towards a downstream two step reaction employing maltase and α-glucosidase instead of a single step α-glucosidase based reaction. The yielded enzyme activity of the target amylase was quantitatively determined by conversion with its specific activity.

## Competing interests

The authors declared that they have no competing interests.

## Authors’ contributions

TK coordinated the study, carried out most of the experimental work, analyzed all results and wrote the paper. VS generated various strains and participated in bioreactor cultivations. Plasmids, promoters and target enzymes were selected by JB and TK. HH, JB, TK and WW participated in the design of the study. In addition to this, HH, JB, WW and TOC offered general and expert supervision. WW drafted and co-wrote the manuscript. KHM conceived the research project on *B. pumilus* and offered general and expert supervision during the initial phase. All authors read and approved the final manuscript.

## Supplementary Material

Additional file 1: Appendix S1 Plasmid constructions. **Table S1.** Schedule of primer sets and respective template DNA to generate the presented plasmids via enzyme free cloning. Click here for file

## References

[B1] Van Dijl JM Hecker M *Bacillus subtilis*: from soil bacterium to super-secreting cell factory Microb Cell Fact 2013 12 3 10.1186/1475-2859-12-3 23311580 PMC3564730

[B2] Goddette D Paech C Yang S The crystal structure of the *Bacillus lentus* alkaline protease, subtilisin BL, at 1.4 Å resolution J Mol Biol 1992 228 580 595 10.1016/0022-2836(92)90843-9 1453465

[B3] Maurer K-H Detergent proteases Curr Opin Biotechnol 2004 15 330 334 10.1016/j.copbio.2004.06.005 15296930

[B4] Priest FG Extracellular enzyme synthesis in the genus Bacillus Bacteriol Rev 1977 41 711 753 334155 10.1128/br.41.3.711-753.1977 PMC414021

[B5] Gupta R Beg Q Lorenz P Bacterial alkaline proteases: molecular approaches and industrial applications Appl Microbiol Biotechnol 2002 59 15 32 10.1007/s00253-002-0975-y 12073127

[B6] Gupta R Beg Q Khan S Chauhan B An overview on fermentation, downstream processing and properties of microbial alkaline proteases Appl Microbiol Biotechnol 2002 60 381 395 10.1007/s00253-002-1142-1 12466877

[B7] Liu L Liu Y Shin H Chen RR Wang NS Li J Du G Chen J Developing *Bacillus spp.* as a cell factory for production of microbial enzymes and industrially important biochemicals in the context of systems and synthetic biology Appl Microbiol Biotechnol 2013 97 6113 6127 10.1007/s00253-013-4960-4 23749118

[B8] Schallmey M Singh A Ward OP Developments in the use of Bacillus species for industrial production Can J Microbiol 2004 50 1 17 10.1139/w03-076 15052317

[B9] Biedendieck R Borgmeier C Bunk B Stammen S Scherling C Meinhardt F Wittmann C Jahn D Systems biology of recombinant protein production using *Bacillus megaterium* Methods Enzymol 2011 500 165 195 21943898 10.1016/B978-0-12-385118-5.00010-4

[B10] Malten M Biedendieck R A *Bacillus megaterium* plasmid system for the production, export, and one-step purification of affinity-tagged heterologous levansucrase from growth medium Appl Environ Microbiol 2006 72 1677 1679 10.1128/AEM.72.2.1677-1679.2006 16461726 PMC1392972

[B11] Garcia-Albornoz MA Nielsen J Application of Genome-Scale Metabolic Models in Metabolic Engineering Ind Biotechnol 2013 9 203 214 10.1089/ind.2013.0011

[B12] Dauner M Sauer U Stoichiometric growth model for riboflavin-producing *Bacillus subtilis* Biotechnol Bioeng 2001 76 132 143 10.1002/bit.1153 11505383

[B13] Wemhoff S Meinhardt F Generation of biologically contained, readily transformable, and genetically manageable mutants of the biotechnologically important *Bacillus pumilus* Appl Microbiol Biotechnol 2013 97 7805 7819 10.1007/s00253-013-4935-5 23644770

[B14] Gioia J Yerrapragada S Qin X Jiang H Paradoxical DNA repair and peroxide resistance gene conservation in *Bacillus pumilus* SAFR-032 PLoS One 2007 2 e928 10.1371/journal.pone.0000928 17895969 PMC1976550

[B15] Link L Sawyer J Venkateswaran K Nicholson W Extreme spore UV resistance of *Bacillus pumilus* isolates obtained from an ultraclean Spacecraft Assembly Facility Microb Ecol 2004 47 159 163 10.1007/s00248-003-1029-4 14502417

[B16] Nahrstedt H Waldeck J Gröne M Eichstädt R Feesche J Meinhardt F Strain development in *Bacillus licheniformis*: construction of biologically contained mutants deficient in sporulation and DNA repair J Biotechnol 2005 119 245 254 10.1016/j.jbiotec.2005.04.003 15951041

[B17] Wilson CR Bacillus cereus plasmid pBC16, complete sequence (GenBank: U32369.1) 1995 2331 Circadian Way, Santa Rosa, CA 95407-5415, USA: COGNIS, Inc., Bioproducts

[B18] Sarvas M Harwood CR Bron S van Dijl JM Post-translocational folding of secretory proteins in Gram-positive bacteria Biochim Biophys Acta 2004 1694 311 327 15546674 10.1016/j.bbamcr.2004.04.009

[B19] Çalik P Bilir E Çalik G Özdamar TH Bioreactor operation parameters as tools for metabolic regulations in fermentation processes: influence of pH conditions Chem Eng Sci 2003 58 759 766 10.1016/S0009-2509(02)00605-X

[B20] Çalik P Tomlin GC Oliver SG Özdamar TH Overexpression of a serine alkaline protease gene in *Bacillus licheniformis* and its impact on the metabolic reaction network Enzym Microb Technol 2003 32 706 720 10.1016/S0141-0229(03)00030-9

[B21] Hübner U Bock U Schügerl K Production of alkaline serine protease subtilisin Carlsberg by *Bacillus licheniformis* on complex medium in a stirred tank reactor Appl Microbiol Biotechnol 1993 40 182 188

[B22] Xiao Z Qiao S Ma C Xu P Acetoin production associated with the increase of cell biomass in *Bacillus pumilus* ATCC 14884 Afr J Biotechnol 2010 4 1997 2003

[B23] McKenzie T Hoshino T Tanaka T Sueoka N The nucleotide sequence of pUB110: some salient features in relation to replication and its regulation Plasmid 1986 15 93 103 10.1016/0147-619X(86)90046-6 3010356

[B24] McKenzie T Hoshino T Tanaka T Sueoka N Correction. A revision of the nucleotide sequence and functional map of pUB110 Plasmid 1987 17 83 85 10.1016/0147-619X(87)90015-1 3033723

[B25] Voigt B Schweder T Sibbald MJJB Albrecht D Ehrenreich A Bernhardt J Feesche J Maurer K-H Gottschalk G van Dijl JM Hecker M The extracellular proteome of *Bacillus licheniformis* grown in different media and under different nutrient starvation conditions Proteomics 2006 6 268 281 10.1002/pmic.200500091 16317772

[B26] Kawamura F Doi RH Construction of a *Bacillus subtilis* double mutant deficient in extracellular alkaline and neutral proteases J Bacteriol 1984 160 442 444 6434524 10.1128/jb.160.1.442-444.1984 PMC214740

[B27] Maier U Büchs J Characterisation of the gas-liquid mass transfer in shaking bioreactors Biochem Eng J 2001 7 99 106 10.1016/S1369-703X(00)00107-8 11173296

[B28] Sambrook J Green MR Molecular Cloning: A Laboratory Manual 2012 3 4 Cold Spring Harbor, New York: Cold Spring Harbor Laboratory Press 2028

[B29] Tillett D Neilan B Enzyme-free cloning: a rapid method to clone PCR products independent of vector restriction enzyme sites Nucleic Acids Res 1999 27 e26 10.1093/nar/27.19.e26 10481038 PMC148636

[B30] Matsumoto A Itoh TQ Self-assembly cloning: a rapid construction method for recombinant molecules from multiple fragments Biotechniques 2011 51 55 56 21781054 10.2144/000113705

[B31] Chang S Cohen SN High frequency transformation of *Bacillus subtilis* protoplasts by plasmid DNA Mol Gen Genet 1979 168 111 115 10.1007/BF00267940 107388

[B32] Lederberg J Lederberg EM Replica plating and indirect selection of bacterial mutants J Bacteriol 1952 63 399 406 14927572 10.1128/jb.63.3.399-406.1952 PMC169282

[B33] Aerobic Plate Count [http://www.fda.gov/food/foodscienceresearch/laboratorymethods/ucm063346.htm]

[B34] Van Raay HG Zur Bestimmung der proteolytischen Aktivität in Enzymkonzentraten und enzymhaltigen Wasch-, Spül-, und Reinigungsmitteln Tenside 1970 7 125 132

[B35] Kruse-Jarres JD Kaiser C Hafkenscheid JC Hohenwallner W Stein W Bohner J Klein G Poppe W Rauscher E Evaluation of a new alpha-amylase assay using 4.6-ethylidene-(G7)-1-4-nitrophenyl-(G1)-alpha-D-maltoheptaoside as substrate Eur J Clin Chem Clin Biochem 1989 27 103 113 2787387

