# Supplementary material for: Developing a new production host from a blueprint: Bacillus pumilus as an industrial enzyme producer
Source: Microb Cell Fact. 2014 Mar 24;13:46. doi: 10.1186/1475-2859-13-46 (PMC3987833; doi:10.1186/1475-2859-13-46)
Supplement: Additional file 1: Appendix S1 — Plasmid constructions. Table S1. Schedule of primer sets and respective template DNA to generate the presented plasmids via enzyme free cloning. [file 1475-2859-13-46-S1.doc]

**Additional file 1**

**Appendix S1: Plasmid constructions.**

Protease BL18 expressing plasmids pHP49 (5.5 kb), pHP59 (4.3 kb), pPB49 (5.5 kb), pHP17 (4.4 kb) and the α-amylase expressing plasmid pHP5-31 (5.7 kb) were provided by Henkel AG & Co. KGaA.

Plasmids constructed in this contribution (pVS13, pVS14, pVS23, pVS24, pVS19, pVS20, pTK5, pTK6, pTK7) were generated by enzyme free cloning [1]. Table S1 gives an overview of the primer sets used to generate the respective PCR-products. The corresponding four different PCR products per cloning experiment were pooled equimolarly for the subsequent hybridization reaction as described by Tillett and Neilan [1]. Subsequently, *B. subtilis* DB104 protoplasts were transformed with the hybridization product. Plasmid DNA isolated from DB104 clones was *in vitro* methylated and used to transform *B. pumilus* Jo2.1 cells.

Reference

1. Tillett D, Neilan B: **Enzyme-free cloning: a rapid method to clone PCR products independent of vector restriction enzyme sites.** *Nucleic Acids Res* 1999, **27**:e26.

Table S1: Schedule of primer sets and respective template DNA to generate the presented plasmids via enzyme free cloning. PCR products of backbones or inserts are consecutively numerically or alphabetically labeled.

Table S1, continued

| **Plasmid**  (size) | **template** | **PCR product** | | **Primer** |
| --- | --- | --- | --- | --- |
| pVS13  (5.8 kb) | P*_apr_*_E1_ (genomic DNA) | insert | A | P-pVS13_for_long |
|  |  |  |  | P-pVS13/14_rev_short |
|  |  |  | B | P-pVS13_for_short |
|  |  |  |  | P-pVS13/14_rev_long |
|  | pHP49 | backbone | 1 | P- pVS13/14_RG_for_long |
|  |  |  |  | P- pVS13/14_RG_rev_short |
|  |  |  | 2 | P- pVS13/14_RG_for_short |
|  |  |  |  | P- pVS13_RG_rev_long |
| pVS14  (5.6 kb) | P*_apr_*_E1_ (genomic DNA) | insert | C | P- pVS14_for_long |
|  |  |  |  | P-pVS13/14_rev_short |
|  |  |  | D | P- pVS14_for_short |
|  |  |  |  | P-pVS13/14_rev_long |
|  | pHP49 | backbone | 1 | P- pVS13/14_RG_for_long |
|  |  |  |  | P- pVS13/14_RG_rev_short |
|  |  |  | 3 | P- pVS13/14_RG_for_short |
|  |  |  |  | P- pVS14_RG_rev_long |
| pVS23  (5.6 kb) | P*_apr_*_E1_ (genomic DNA) | insert | E | P-pVS23_for |
|  |  |  |  | P-pVS13/14_rev_short |
|  |  |  | F | P-pVS23_for_short |
|  |  |  |  | P-pVS13/14_rev_long |
|  | pHP49 | backbone | 4 | P- pVS13/14_RG_for_long |
|  |  |  |  | P-pVS23_rev_short |
|  |  |  | 5 | P- pVS13/14_RG_for_short |
|  |  |  |  | P-pVS23_rev |
| pVS24  (5.7 kb) | P*_apr_*_E1_ (genomic DNA) | insert | G | P-pVS24_for |
|  |  |  |  | P-pVS13/14_rev_short |
|  |  |  | H | P-pVS24_for_short |
|  |  |  |  | P-pVS13/14_rev_long |
|  | pHP49 | backbone | 6 | P- pVS13/14_RG_for_long |
|  |  |  |  | P-pVS24_rev_short |
|  |  |  | 7 | P- pVS13/14_RG_for_short |
|  |  |  |  | P-pVS24_rev |
| pVS19  (5.5 kb) | P*_mpr_* (genomic DNA) | insert | I | P-pVS19_for_long |
|  |  |  |  | P- pVS19_rev_short |
|  |  |  | J | P- pVS19_for_short |
|  |  |  |  | P- pVS19_rev_long |
|  | pHP49 | backbone | 8 | P-pVS18_RG_for_long |
|  |  |  |  | P-pVS18_RG_rev_short |
|  |  |  | 9 | P-pVS18_RG_for_short |
|  |  |  |  | P-pVS18_RG_rev_long |

| **Plasmid** | **template** | **PCR product** | | **Primer** |
| --- | --- | --- | --- | --- |
| pVS20  (5.6 kb) | P*_apr_*_E2_ (genomic DNA) | insert | K | P-pVS20_for_long |
|  |  |  |  | P-pVS20_rev_short |
|  |  |  | L | P-pVS20_for_short |
|  |  |  |  | P-pVS20_rev_long |
|  | pHP49 | backbone | 8 | P-pVS18_RG_for_long |
|  |  |  |  | P-pVS18_RG_rev_short |
|  |  |  | 9 | P-pVS18_RG_for_short |
|  |  |  |  | P-pVS18_RG_rev_long |
| pTK5  (5.7 kb) | P*_mpr_* (genomic DNA) | insert | M | pTK5/6/7_Insert_for_long |
|  |  |  |  | pTK5_Insert_rev_short |
|  |  |  | N | pTK5/6/7_Insert_for_short |
|  |  |  |  | pTK5_Insert_rev_long |
|  | pHP5-31 | backbone | 10 | pHP5-31_backbone_for_long |
|  |  |  |  | pHP5-31_backbone_rev_short |
|  |  |  | 11 | pHP5-31_backbone_for_short |
|  |  |  |  | pHP5-31_backbone_rev_long |
| pTK6  (5.8 kb) | P*_apr_*_E1_ (genomic DNA) | insert | O | pTK5/6/7_Insert_for_long |
|  |  |  |  | pTK6_Insert_rev_short |
|  |  |  | P | pTK5/6/7_Insert_for_short |
|  |  |  |  | pTK6_Insert_rev_long |
|  | pHP5-31 | backbone | 10 | pHP5-31_backbone_for_long |
|  |  |  |  | pHP5-31_backbone_rev_short |
|  |  |  | 11 | pHP5-31_backbone_for_short |
|  |  |  |  | pHP5-31_backbone_rev_long |
| pTK7  (5.8 kb) | P*_apr_*_E2_ (genomic DNA) | insert | Q | pTK5/6/7_Insert_for_long |
|  |  |  |  | pTK7_Insert_rev_short |
|  |  |  | R | pTK5/6/7_Insert_for_short |
|  |  |  |  | pTK7_Insert_rev_long |
|  | pHP5-31 | backbone | 10 | pHP5-31_backbone_for_long |
|  |  |  |  | pHP5-31_backbone_rev_short |
|  |  |  | 11 | pHP5-31_backbone_for_short |
|  |  |  |  | pHP5-31_backbone_rev_long |
